# A classical variant of ectodermal dysplasia: a case report

**DOI:** 10.11604/pamj.2025.52.90.48271

**Published:** 2025-11-03

**Authors:** Switi Jawade, Pratibha Wankhede, Ranjana Sharma, Pratiksha Munjewar, Vidhya More

**Affiliations:** 1 Department of Obstetrics and Gynecology Nursing, Shalinitai Meghe College of Nursing, Salod (Hirapur), Datta Meghe Institute of Higher Education and Research (Deemed to be University), Sawangi, Wardha, Maharashtra, India; 2 Department of Community Health nursing, Shalinitai Meghe College of Nursing, Salod (Hirapur), Datta Meghe Institute of Higher Education and Research (Deemed to be University), Sawangi, Wardha, Maharashtra, India; 3 Department of Medical Surgical Nursing, Shalinitai Meghe College of Nursing, Salod (Hirapur), Datta Meghe Institute of Higher Education and Research (Deemed to be University), Sawangi, Wardha, Maharashtra, India

**Keywords:** Ectodermal dysplasia, genetic disorder, hypodontia, hypohidrosis, case report

## Abstract

Ectodermal dysplasias are characterized by abnormalities in two or more ectodermal structures, including hair, sweat glands, nails, etc. and are a rare group of congenital disorders. It is characterized by the triad of three conditions, namely hypohidrosis, hypotrichosis, and hypodontia. The phenotypic expression of ectodermal dysplasia may be variable, often raising problems in clinical assessments and care, particularly in the paediatric age group, where early manifestations can be subtle or nonspecific. While most cases reveal a clear inheritance pattern, sporadic presentations in the absence of similar complaints in the family are uncommon and raise significant diagnostic difficulties. Early identification and multidisciplinary treatment are imperative to mitigate complications such as recurrent infections, thermoregulatory dysfunction, and psychosocial impact. In this report, we highlight the clinical presentation, diagnostic evaluation, and management considerations in a 10-year-old male child diagnosed with a sporadic case of ectodermal dysplasia.

## Introduction

Ectodermal dysplasia is an uncommon group of genetic disorders that mainly exhibit developmental anomalies. Congenital abnormalities in two or more ectodermal structures, such as the skin, nails, teeth, hair, or sweat glands, are its defining feature. The underlying etiology involves genetic mutations that disrupt key molecular pathways responsible for the differentiation, signalling, and morphogenesis of ectodermal tissues during embryogenesis. During this critical developmental phase, the ectoderm forms the outermost layer and gives rise to structures that are most commonly affected in ectodermal dysplasia.

There are over 150 variations of ectodermal dysplasia (ED) documented in the literature, and its incidence is 7 per 10,000 live births [1]. Genetic mutations implicated in ectodermal dysplasia commonly involve the ectodysplasin A, ectodysplasin A Receptor, and ectodysplasin A receptor-associated death domain genes, among others, which play essential roles in the ectodysplasin signalling pathway crucial for ectodermal organ development. The X-linked recessive Mendelian trait known as hereditary hypohidrotic ectodermal dysplasia is the most common type and is typically found in males and is passed down through female carriers. While females only exhibit modest abnormalities, males are severely affected [2]. Hypohidrotic form presents with a triad of hypotrichosis, hypodontia, and hypohidrosis. Early recognition is crucial, especially in children, to reduce adverse effects like heat intolerance and to initiate timely multidisciplinary care.

The disease generally has three clinical variants: hypohidrotic/anhidrotic ectodermal dysplasia also known as Christ-Siemens-Touraine syndrome, has either completely absent or drastically reduced sweat glands, it has an X-linked pattern of inheritance is caused by mutations in EDA1 gene which is located on the X chromosome [3], hidrotic or Clouston syndrome, clinically presents with hypotrichosis, palmoplantar hyperkeratosis and nail dystrophy, although has normal sweat glands. It has an autosomal dominant pattern of inheritance and is caused by mutations in the Gap Junction beta 6 gene [4]. Wiktop tooth and nail syndrome is a rare disorder having an autosomal dominant inheritance pattern, which is caused by a nonsense mutation in the muscle segment homeobox 1 gene, and it is characterized by hypodontia and nail dysplasia [5].

## Patient and observation

**Figure 1:**
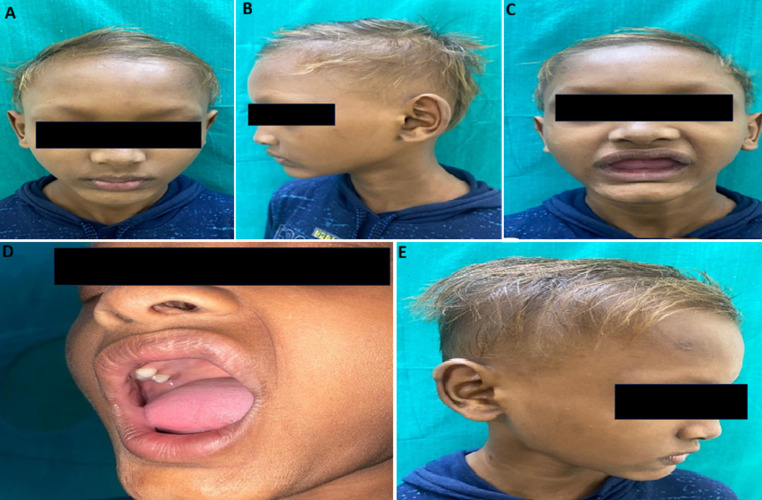
A) sparse scalp and eyebrows; B) hypotrichosis of eyebrows; C) absence of teeth (hypodontia); D) dental anomalies with conoid teeth; E) showing depressed nasal bridge

**Figure 2:**
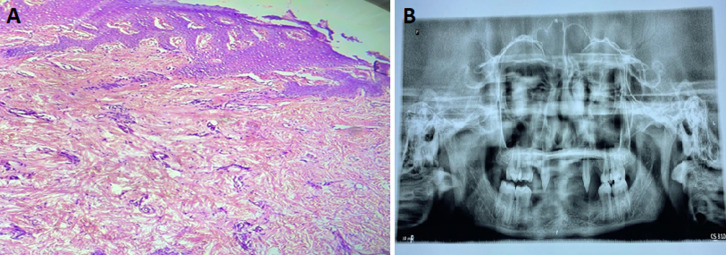
A) reduced number of sebaceous glands and complete absence of sweat glands; B) oligodontia and peg-shaped teeth

**Patient information:** a 10-year-old male child, accompanied by his father, came to the outpatient department of dermatology with a complaint of decreased sweating all over the body and recurrent episodes of upper respiratory tract infections since birth. He also gave a history of sparseness of hair over the scalp and eyebrows, delayed eruption of teeth, and partial absence of teeth for his age. His father also reported that the child had pronounced heat intolerance, fatigue, and episodes of nasal bleeding in extreme heat. He also complained of hoarseness of voice. The child was born out of a non-consanguineous marriage, and there was no history of similar complaints in the family (Figure 1, Figure 2).

**Clinical findings:** upon thorough clinical assessment, the patient´s vital parameters were found to be within normal physiological ranges, and a comprehensive systemic evaluation revealed no detectable alterations. Dermatological examination revealed scanty, fragile, light brown coloured hair on the scalp and notably sparse eyebrows. The patient exhibited characteristic craniofacial features such as frontal bossing, periorbital wrinkling, and a saddle nose with a depressed nasal bridge. The skin over the trunk was warm, dry, with a complete absence of hair on both upper and lower extremities. Intraoral examination revealed dry mucosa, multiple missing teeth in both mandibular and maxillary arches, and the teeth that were present were peg-shaped. Nails appeared normal, and there was no evident hand or foot deformity.

Timeline for current episode

**Diagnostic assessment:** on performing routine investigations, complete blood count was normal, and PS showed microcytic hypochromic red blood cells. To assess the functioning of the sweat glands, a series of bedside tests was conducted. The starch Iodine test was performed, where a thin layer of tincture iodine was applied on the right axilla of the patient, followed by dusting with corn starch powder. The absence of any colour change indicated the absence of sweating.

A hair mount test was also carried out to evaluate hair morphology. The patient's hair shaft was compared to that of a normal hair of a child of similar age. Light microscopy revealed scant and thin hair. The cortex was thin and irregular, and the medulla was nearly absent. To further assess sweat gland activity, a pilocarpine test was performed on the left forearm. Pilocarpine 0.2 ml was injected intradermally on the left forearm. After a few minutes, the area showed unchanged colour, suggesting an absent sweat response. A skin biopsy was taken from the hypothenar eminence of the right palm using a 3 mm punch. Histopathological examination revealed a reduced number of sebaceous glands and a complete absence of sweat glands. Additionally, an orthopantomogram was performed, which showed oligodontia and peg-shaped teeth in the maxillary arch.

**Therapeutic Intervention and outcome:** antibiotic azithromycin was prescribed, the physician advised to avoid heat exposure, frequent consumption of cool liquid, and also advised to wear light, loose-fitting clothes. Carefully monitored for body temperature. Orthodontic treatment was going on, and a plan for prosthodontic rehabilitation in the future. This helps to control the symptoms and may improve the child's quality of life.

**Patient perspective:** a child's father was satisfied with the treatment management by a physician.

**Informed consent:** written informed consent was taken from the child's parents for publication of this case report.

## Discussion

Ectodermal dysplasia represents a heterogeneous group of disorders characterized by the abnormal development of two or more ectodermal structures-primarily hair, teeth, nails, and sweat glands. This case report presents a 10-year-old male child diagnosed with hypohidrotic ectodermal dysplasia, also known as Christ-Siemens-Touraine Syndrome, a classical variant of ectodermal dysplasia.

The child exhibited hallmark features of hypohidrotic ectodermal dysplasia, including sparse scalp and eyebrow hair, hypohidrosis, and significant dental anomalies such as delayed tooth eruption and hypodontia. Additionally, the patient demonstrated heat intolerance since early childhood, resulting in recurrent episodes of hyperthermia, particularly exacerbated by physical exertion and high environmental temperatures, reflecting the compromised function of sweat glands. Recurrent upper respiratory tract infections, epistaxis on heat exposure were also notable, and hoarseness of voice were also noted, suggesting mucosal involvement due to atrophic or dysfunctional glandular structures.

A detailed family history revealed no similar symptoms in siblings, reinforcing the suspicion of a sporadic inheritance pattern, which is an uncommon presentation. The child was born of a non-consanguineous union and displayed normal growth, intelligence, and developmental milestones, suggesting the ectodermal defects were localized without systemic neurodevelopmental compromise.

Investigations, including pilocarpine-induced sweat testing and hair microscopy, substantiated the clinical diagnosis. Skin biopsy, though not routinely performed, provided supportive histological evidence in this case by confirming eccrine gland aplasia-a defining pathological feature of hypohidrotic ectodermal dysplasia [6]. Histopathology and radiological evaluation with orthopantomogram confirmed dental agenesis. The findings are consistent with a mutation in the ectodysplasin-A gene, pivotal in the EDA signaling pathway, which governs the morphogenesis of ectodermal derivatives. Disruption of this pathway leads to varying degrees of aplasia or dysplasia of affected tissues.

Management was tailored to address both physiological and psychosocial needs. Dermatological care focused on skin hydration and sun protection, while thermoregulatory cooling strategies to prevent overheating, which is a common and potentially serious concern in patients with anhidrosis [7], were emphasized to prevent heat-related complications. Speech therapy was advised in view of the hoarseness of the voice. Prosthetic dental rehabilitation was advised once the child reached 18 years of age to improve masticatory function and aesthetics [8]. Psychosocial support and multidisciplinary follow-up were integral to ensuring quality of life and holistic well-being.

## Conclusion

This case underscores the importance of early clinical recognition of hypohidrotic ectodermal dysplasia, especially in children presenting with the triad of sparse hair, missing teeth, and impaired sweating. Histopathological and diagnostic adjuncts like sweat testing and imaging can aid confirmation. While our case presented with typical features of hypohidrotic ectodermal dysplasia, the rare occurrence of complaints without any family member being affected makes it a rare sporadic presentation pattern. Management should be multidisciplinary, aiming at symptomatic relief, psychological support, and genetic counselling. Early intervention not only mitigates complications but also enhances the quality of life.
